# Complete genome sequence of a *Staphylococcus condimenti* isolated from a port catheter-associated bloodstream infection

**DOI:** 10.1128/MRA.00438-23

**Published:** 2023-09-01

**Authors:** Laura Berneking, Henning Büttner, Minyue Qi, Thomas Günther, Daniel Lehnhoff, Anna Both, Martin Christner, Manuel Wolters, Malik Alawi, Martin Aepfelbacher, Holger Rohde

**Affiliations:** 1 Institute for Medical Microbiology, Virology and Hygiene, University Medical Center Hamburg-Eppendorf, Hamburg, Germany; 2 Bioinformatics Core, University Medical Center Hamburg-Eppendorf, Hamburg, Germany; 3 Leibniz Institute of Virology, Hamburg, Germany; 4 MVZ Labor Dr. Fenner, Hamburg, Germany; University of Rochester School of Medicine and Dentistry, Rochester, New York, USA

**Keywords:** coagulase-negative staphylococci, *Staphylococcus condimenti*, bloodstream infection

## Abstract

Here, we describe the complete genome sequence of a *Staphylococcus condimenti* blood culture isolate from a catheter-related bloodstream infection in a male patient.

## ANNOUNCEMENT

*Staphylococcus condimenti* was first described in 1998 as a subgroup of *Staphylococcus carnosus* (1), a coagulase-negative staphylococcus (CoNS) employed in food starter cultures for the characteristic flavor of sausages and cured ham (2). CoNS relevant in food processing, including *S. condimenti*, are rarely associated with human disease (3–7).

We describe the full-genome sequence of a *S. condimenti* isolated in 2010 in Hamburg, Germany, from a 42-year-old male patient presenting with a port catheter-related bloodstream infection. *S. condimenti* was isolated from five independent blood cultures (BACTEC PLUS, BD, Heidelberg, Germany), incubated for a maximum of 5 days, and from the explanted port catheter system. Blood cultures were streaked onto Columbia blood agar (Oxoid), incubated at 37°C overnight in ambient air, and colonies were identified to the species level by whole-cell MALDI-TOF fingerprinting (Biotyper) and partial 16S ribosomal RNA gene sequencing (8), showing 100% identity of non-ambiguous bases to the 16S rRNA of type strain *S. condimenti* DSM11674T. Antibiotic susceptibility was determined using gradient strips (Liofilchem), following the manufacturer’s instructions. All isolates were susceptible to penicillin (MIC 0.064 µg/mL), oxacillin (MIC 0.5 µg/mL), vancomycin (MIC 1.0 µg/mL), clindamycin (MIC 0.064 µg/mL), clarithromycin (MIC 0.125 µg/mL), and linezolid (MIC 1.0 µg/mL), according to EUCAST breakpoints.

One *S*. *condimenti* isolate (*S. condimenti* 6209, obtained from the first positive blood culture) was subjected to short- and long-read sequencing. Genomic DNA was extracted using the QIAsymphony DSP virus/pathogen (for short-read sequencing) and the Blood and Cell Culture DNA Midi Kit (for long-read sequencing; both Qiagen), respectively. The Nextera DNA Kit (Illumina) was used for library construction for paired-end (2 × 151 bp) sequencing on a MiSeq instrument (Illumina). Over two million reads were obtained and subsequently processed with fastp v0.20.1 (9). Default parameters were used for all mentioned software.

For ONT sequencing, 2 µg DNA was fragmented to an average length of 8 kb using Covaris g-Tube and subjected to library preparation using the Nanopore Sequencing Kit (SQN-NSK006). The library was sequenced on a MinION device using the NC_48 hr_Sequencing_Run_FLO-MIN103.py workflow of the MinKnown software, according to the manufacturer’s guidelines. Reads with an average read quality value below 5, according to metrichor cloud-based base calling (metrichor.com), were discarded, resulting in 4,475 reads (read N50: 11,704 bp) subjected to analysis.

A hybrid assembly was performed using Unicycler (v0.5.0) (10). Six contigs with a total length of 2,624,647 bp and a GC content of 34.65% were obtained (N50: 2,617,439 bp, average coverage 216×). Annotation was performed with Prokka (v1.14.5) (11). Comparisons with three publicly available *S. condimenti* assemblies performed with OrthoANIu calculator (12) yielded ANI estimates >99% (Table 1). In agreement with phenotypic testing, CARD resistance gene profiling (https://card.mcmaster.ca/) identified no antibiotic resistance genes.

**TABLE 1:** Average nucleotide identity of *S. condimenti* 6209 compared to available *S. condimenti* genomes

Query isolate	Reference	NCBI RefSeq assembly	SRA raw data	ANI estimate (%)
*S. condimenti* 6209	*S. condimenti* DSM 11674 (13)	GCF_002902505.1	SRR5029747	99.88
*S. condimenti* 6209	*S. condimenti* StO2014-01 (3)	GCF_001922405.1	n/a^*a*^	99.88

^*a*^ not available.

The report adds to evidence (3, 5–7, 14) that *S. condimenti* is able to cause invasive disease in predisposed individuals. Genomic information from invasive isolates may support food safety efforts in the future.

## Data Availability

The genome assembly is available at the NCBI Sequence Read Archive (SRA) under accession PRJNA936100. Sequence reads are available at the European Nucleotide Archive (ENA) under accession PRJEB61960.
